# Associations between reversible and potentially reversible cognitive frailty and falls in community-dwelling older adults in China: a longitudinal study

**DOI:** 10.1186/s12877-025-05872-2

**Published:** 2025-04-05

**Authors:** Xiaonuo Xu, Ning Ding, Jing He, Ronghui Zhao, Weiqi Gu, Xiaoyan Ge, Kai Cui

**Affiliations:** School of Public Health, Jinzhou Medical University, 40 Songpo Road, Jinzhou, 121000 P. R. China

**Keywords:** Cognitive impairment, Frailty, Falls, Older adults

## Abstract

**Background:**

Few studies have focused on comparing the effect of cognitive frailty (CF) with either cognitive impairment or frailty alone on fall risk. Further, studies investigating the effect of reversible cognitive frailty (RCF) or potentially reversible cognitive frailty (PRCF) on fall risk are scarce. This study aimed to investigate the influence of RCF and PRCF on falls in community-dwelling older adults of China and determine whether CF conferred a higher risk than cognitive impairment or frailty alone.

**Methods:**

This study used data from five waves of the China Health and Retirement Longitudinal Study (CHARLS) conducted from 2011 to 2020. A total of 3,200 participants were divided into six groups: Healthy, cognitive impairment [subjective cognitive decline (SCD) and mild cognitive impairment (MCI)], Frailty, and CF (RCF and PRCF), according to their baseline cognitive and frailty status. A generalized estimating equation was applied to measure the association of cognitive status, frailty, and CF with risk of falls. Multivariate logistic regression models were employed to analyze potential multiplicative and additive interactions of baseline cognitive impairment and frailty on fall risk.

**Results:**

Of the 3,200 participants, 17.7% and 8.3% experienced falls and fall-induced injuries, respectively, in wave 2013. After adjusting for all covariates, the participants in the PRCF group [odds ratio (OR) = 1.442, 95% confidence interval (CI): 1.179–1.922] had a higher risk of falling than those in the RCF group (OR = 1.302, 95% CI: 1.053–1.593), while cognitive impairment alone or frailty alone were not associated with increased risk. The interaction analyses revealed a lack of multiplicative (OR *=* 0.952, 95% CI: 0.618–1.468) or additive [relative excess risk (RERI) =-0.043, 95% CI: -0.495–0.409; attributable proportion (AP) =-0.035, 95% CI: -0.400–0.329; synergy index (S) *=* 0.840, 95% CI: 0.172–4.095] interactions of cognitive impairment and frailty for falls.

**Conclusions:**

We found that the risk of falls increased in RCF and PRCF compared to either cognitive impairment or frailty alone, with PRCF being associated with a higher risk than RCF.

**Clinical trial number:**

Not applicable.

**Supplementary Information:**

The online version contains supplementary material available at 10.1186/s12877-025-05872-2.

## Background

The global population is aging rapidly, with China currently having the largest number of older adults worldwide; this figure only continues to rise [1]. Population aging thus poses considerable challenges to healthcare in China, as falls have become the primary cause of death among adults aged 60 years and older, significantly increasing the burden on healthcare systems [1–3]. The World Health Organization defined falls as accidental falls on the ground, excluding falls due to ongoing violence or seizure [4]. The global prevalence of falls among older adults is reported to be 26.5% [5]. In China, approximately 30% of older adults (~ 50 million individuals) experience falls each year [6].

In 2013, the International Academy of Nutrition and Aging and the International Association of Gerontology and Geriatrics introduced diagnostic criteria for cognitive frailty (CF), defining it as the coexistence of physical frailty and cognitive impairment in older adults without a definite diagnosis of dementia [7]. Frailty is an age-related pathophysiological state of homeostasis and stress resistance, and individuals can be divided into non-frailty, pre-frailty, or frailty groups [8–11]. Cognitive impairment is defined as a decline in subjective and objective functions in one or more cognitive dimensions, without severely affecting daily activities or mental diseases [12]. Prior studies have shown that CF can cause disability; reduce the quality of life; and increase the risk of falls due to impairments in executive function, attention, and responses to hazardous situations [13, 14]. Several studies have reported that falls occur in 40% of older adults who experience CF [15], with these adults having at least double the risk compared to those without CF [16–18]. However, whether CF is associated with a greater or lesser risk of falls compared to cognitive impairment or frailty alone is still controversial. In one study from the United States, the risk of falls in older adults with CF was increased compared to that in individuals with either cognitive impairment or frailty alone [19]. Conversely, Ma et al. observed that the risk of falls in older Chinese adults with frailty was higher than in those with CF [18]. Therefore, it is necessary to compare the effect of CF on the risk of falls in individuals with cognitive impairment or frailty alone.

There are two CF subtypes: reversible cognitive frailty (RCF) and potentially reversible cognitive frailty (PRCF) [20]. RCF is defined as the co-occurrence of frailty or pre-frailty and subjective cognitive decline (SCD) without acute impairment or a clinical diagnosis of neurodegenerative or other mental-health conditions; SCD refers to a self-reported perception of memory decline despite normal cognitive assessment results [21, 22]. PRCF is defined as the co-occurrence of physical frailty or pre-frailty and mild cognitive impairment (MCI) [20]. The term MCI describes a state of memory and thinking difficulties with lower-than-normal scoring averages in objective neurocognitive scales [15]. At present, few studies have focused on the effect of RCF or PRCF on fall risk.

Herein, we conducted a longitudinal study to determine whether CF increases the risk of falls compared to cognitive impairment or frailty alone and to investigate the effects of RCF and PRCF on fall incidence. This study thus aimed to provide a scientific basis for identifying high-risk individuals and developing effective fall prevention strategies among older adults.

## Methods

### Data collection and study participants

The China Health and Retirement Longitudinal Study (CHARLS) is a representative longitudinal survey of individuals aged 45 years and older in mainland China. The national baseline survey was conducted from 2011 to 2012, with four waves of routine questionnaires administered in 2013, 2015, 2018, and 2020. To ensure that the data were obtained from a representative sample, CHARLS covered 150 regions and 450 villages or urban communities, and enrolled 17,708 people from 10,257 households, reflecting the demographic situation of middle-aged and older adults in China [23]. All collected data were stored at the China Social Science Survey Center at Peking University, and survey data were publicly published on the website (https://charls.pku.edu.cn) [24]. This longitudinal study was approved by the Ethical Review Committee of Peking University (IRB00001052-11015), and all participants provided informed consent to participate.

**Fig. 1:**
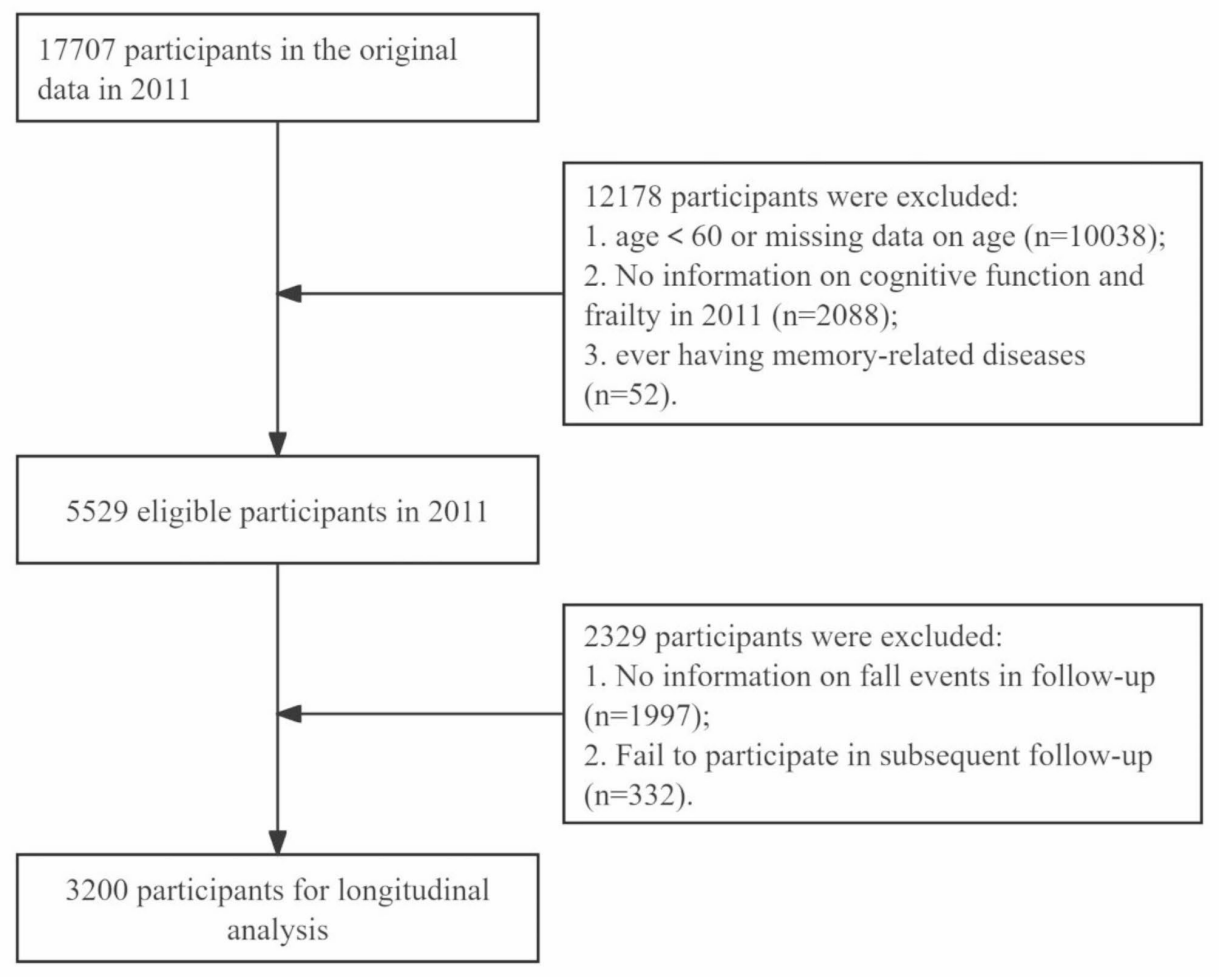
Flow chart of the analytic process of sample collection

This study utilized data from all five waves of CHARLS (2011, 2013, 2015, 2018, and 2020). Wave 2011 served as the baseline, while the subsequent waves (2013–2020) represented the follow-up period. The inclusion criteria were participants aged 60 years and older. The exclusion criteria were as follows: (1) Missing information on age, cognitive function, and frailty in 2011, with lack of information on two or more of the five components of the frailty assessment being counted as no information on frailty [25]; (2) history of memory-related diseases at baseline, such as Alzheimer’s or Parkinson’s disease; (3) missing information on fall events in waves 2013–2020; (4) failure to participate in subsequent follow-up. Overall, we enrolled 3,200 participants and a flowchart detailing the sample selection process is shown in Fig. 1.

### Assessment of frailty

Frailty was evaluated using the Fried physical frailty phenotype (PFP) framework, which is based on five criteria: slowness, weakness, exhaustion, inactivity, and shrinking [9]. The level of frailty was determined depending on the number of criteria met. Participants not meeting any criteria were considered with “non-frailty;” those meeting one or two, with “pre-frailty;” and those meeting three to five, with “frailty.” This method has previously been validated for estimation of frailty prevalence in older Chinese adults using CHARLS data [26]. In this study, both frailty and pre-frailty were categorized as frailty.

Slowness: The mean speed was calculated in two walking speed tests over a 2.5-m distance. Slowness was defined as mean speed equal to or below the 20th percentile after adjusting for participant sex and height via linear regression models [26].

Weakness: The maximum grip strength of the dominant hand was measured twice using a grip meter. Weakness was defined if the average of the two measurements was at or below the 20th percentile of the population distribution after adjusting for sex and body mass index (BMI) using linear regression models [26].

Exhaustion: The criterion was based on responses to two questions from the modified Center for Epidemiological Studies-Depression Scale (CES-D) regarding feelings of effort in the past week: “How often did you feel like ‘I can’t start’ or ‘I feel like everything I’m doing is effortful’ during the last week?” [27]. Responses of “occasionally or moderately (3–4 days)” or “mostly or all of the time (5–7 days)” were determined to indicate exhaustion [26].

Inactivity: Reports of no walking or moderate physical activity for at least 10 consecutive minutes within a week were considered to indicate inactivity [26].

Shrinking: If a participant’s BMI was 18.5 kg/m^2^ or lower, or the participant experienced a weight loss of 5 kg or more between two waves, he or she was considered to have met this criterion [26].

### Assessment of cognitive impairment

Cognitive function was assessed using methods from the American Health and Retirement Study (HRS) [28]. Four cognitive domains were assessed: orientation, memory, calculation, and drawing [29]. Orientation referred to the identification of the year, month, day, day of the week, and the current season, with a maximum score of 5 (one point for each item) [30]. For calculation, the participants were asked to perform five serial subtractions of seven from 100, earning one point for each correct answer, with a maximum score of 5 [31]. The sum of immediate and delayed word recall was used as the total score of memory performance. The participants were presented with 10 Chinese words and underwent evaluation for immediate word recall by calculating the number of words that could be recalled immediately, with 1 point for each word recalled correctly [32]. Word recall was repeated after the participants completed the remaining tests, and the number of words recalled correctly represented the score of delayed recall. Drawing ability was tested by having the participants reproduce two overlapping images of pentagrams, earning 1 point for each correct drawing [33]. The overall cognitive score ranged from 0 to 31, with higher scores indicating better cognitive function [34]. MCI was defined using the criteria for aging-associated cognitive decline (AACD) as scores below the average minus one standard deviation (SD) after stratification by age [35]. Participants aged 60 years and older were grouped in 5-year intervals, and those who met the AACD criteria in each age group were classified as having MCI. SCD was assessed with a question on current memory quality in the database: “How would you rate your memory at present? Would you say that it is excellent, very good, good, fair, or poor?” Those not meeting the MCI criteria but rating their memory as “fair” or “poor” were classified as experiencing SCD [36]. In our study, both MCI and SCD were considered to indicate cognitive impairment.

### Assessment of fall events

We considered two outcomes: falls and fall-induced injuries requiring medical treatment, as reported in a previous study [37]. The first outcome was assessed in CHARLS using the question, “Have you fallen down during the follow-up time period?” Responses were binary (yes or no). The second outcome was determined by asking, “How many times have you fallen down during the follow-up time period seriously enough to need medical treatment?” The number of fall-induced injuries was subsequently recorded.

### Classification of the groups

The participants were categorized into six groups according to their baseline frailty (non-frailty or frailty) and cognitive status (normal cognition, SCD, or MCI): Healthy (non-frailty and normal cognition), SCD (non-frailty and SCD), MCI (non-frailty and MCI), Frailty (frailty and normal cognition), RCF (frailty and SCD), and PRCF (frailty and MCI).

### Covariates

The potential covariates were age, sex, residential area, educational level, marital status, sleep duration, smoking behavior, alcohol consumption, presence of depressive symptoms, BMI, and number of comorbidities. The educational level was classified as illiteracy, elementary school, middle school, or college and above. Sleep duration was categorized into <6 h, 6–9 h, or ≥ 9 h. BMI was calculated as the body weight (kg) divided by the square of height (m^2^) and classified as underweight (< 18.5), normal (18.5–23.9), overweight (24.0–27.9), or obese (≥ 28.0) [38]. Depression was assessed using the 10-item CES-D scale [39], with a CES-D score ≥ 12 indicating symptoms of depression [40]. To evaluate the number of comorbidities, we considered the presence of physician-diagnosed conditions (hypertension, dyslipidemia, hyperglycemia, cancers, chronic lung diseases, liver diseases, heart diseases, stroke, kidney diseases, digestive diseases, arthritis or rheumatism, and asthma).

### Statistical analyses

The baseline characteristics of each participant group are described. Quantitative data with normal distribution are presented as the mean ± SD, while one-way analysis of variance was used for comparisons among the six groups. Qualitative data are reported as percentages, with chi-square tests evaluating differences among the groups.

Generalized estimating equation (GEE) were used to investigate potential associations of cognitive status, frailty, and CF with risk of falls and fall-induced injuries, expressed as odds ratios (OR) with 95% confidence intervals (CI). We used the unstructured correlation structure as the working correlation structure. Time was used as the categorical variable, with the Healthy group serving as the reference. Model 1 was univariate; Model 2 was adjusted for age and sex; and Model 3 was further adjusted for all covariates. In addition, we assessed the interaction between time and group to examine variability in the risk of falls across different cognitive and frailty states over time.

Multivariate logistic regression models were constructed to analyze the multiplicative and additive interactions between baseline cognitive impairment, frailty, and fall risk. The dependent variable was defined as the occurrence of at least one fall event in the waves 2013–2020. For multiplicative interactions, we constructed a logistic regression model using cognitive impairment, frailty, and product terms. For additive interactions, we transformed cognitive impairment and frailty into three dummy variables. The β and OR values were calculated to compute the additive evaluation index, which included quantifying the relative excess risk (RERI), attributable proportion (AP), and synergy index (S), as reported in a previous study [41]. Additive interaction was indicated if the 95% CI for RERI and AP did not include 0, and the 95% CI for S did not include 1.

Lastly, we determined the relationship between CF and risk of falls and fall-induced injuries across different age and sex subgroups, adjusting for all covariates. The forest plots for the subgroup analysis were plotted using the R packages “grid” and “forestploter.” In the sensitivity analysis, we combined RCF and PRCF into a single CF group and reapplied the GEE model to verify the stability of the results.

All statistical analyses were conducted using the SPSS software version 26.0 and R version 4.3.2, with significance set at *P* < 0.05.

## Results

In wave 2011, the distribution of the population across the six groups was as follows: Healthy, 276 (8.6%); SCD, 872 (27.3%); MCI, 162 (5.1%); Frailty, 180 (5.6%); RCF, 1311 (41.0%); and PRCF, 399 (12.5%). Table 1 presents the characteristics of the study population in wave 2011. The average age of the 3,200 participants was 66.3 ± 5.5 years and 48.8% were men. Among the participants, 567 (17.7%) and 266 (8.3%) experienced falls and fall-induced injuries, respectively, in wave 2013. The PRCF and Healthy groups had the highest and lowest proportion of falls in wave 2013, respectively (Table 1).

**Table 1:** Characteristics of the study population in wave 2011

Variables	Healthy	SCD	MCI	Frailty	RCF	PRCF	*P*-value
Participants, n (%)	276 (8.6)	872 (27.3)	162 (5.1)	180 (5.6)	1311 (41.0)	399 (12.5)	
Age, (years, $\:\stackrel{-}{x}\pm\:s$)	65.5 ± 4.7	65.2 ± 4.5	65.2 ± 4.4	67.1 ± 4.8	66.1 ± 5.0	66.2 ± 5.0	< 0.001
Age group, n (%)							0.001
60–75	255 (92.4)	814 (93.3)	150 (92.6)	155 (86.1)	1157 (88.3)	359 (90.0)	
≥ 75	21 (7.6)	58 (6.7)	12 (7.4)	25 (13.9)	154 (11.7)	40 (10.0)	
Sex, n (%)							< 0.001
Male	167 (60.5)	474 (54.4)	56 (34.6)	105 (58.3)	645 (49.2)	113 (28.3)	
Female	109 (39.5)	398 (45.6)	106 (65.4)	75 (41.7)	666 (50.8)	286 (71.7)	
Residential area, n (%)							< 0.001
Rural	184 (66.7)	655 (75.1)	148 (91.4)	152 (84.4)	1121 (85.5)	379 (95.0)	
Urban	92 (33.3)	217 (24.9)	14 (8.6)	28 (15.6)	190 (14.5)	20 (5.0)	
Educational level, n (%)							< 0.001
Illiteracy	45 (16.3)	206 (23.6)	105 (64.8)	49 (27.2)	393 (30.0)	276 (69.2)	
Elementary school	133 (48.2)	458 (52.5)	51 (31.5)	88 (48.9)	703 (53.6)	119 (29.8)	
Middle school	87 (31.5)	193 (22.1)	6 (3.7)	41 (22.8)	205 (15.6)	4 (1.0)	
College or above	11 (4.0)	15 (1.7)	0 (0.0)	1 (0.6)	10 (0.8)	0 (0.0)	
Marital status, n (%)							0.013
Married	226 (81.9)	744 (85.3)	131 (80.9)	145 (80.6)	1051 (80.2)	309 (77.4)	
Others	50 (18.1)	128 (14.7)	31 (19.1)	35 (19.4)	260 (19.8)	90 (22.6)	
Sleep duration, n (%)							< 0.001
< 6 h	54 (19.6)	252 (28.9)	49 (30.2)	48 (26.7)	498 (38.0)	172 (43.1)	
6–9 h	194 (70.3)	557 (63.9)	95 (58.6)	109 (60.6)	714 (54.5)	186 (46.6)	
≥ 9 h	27 (9.8)	57 (6.5)	15 (9.3)	22 (12.2)	90 (6.9)	36 (9.0)	
Smoking behavior, n (%)							< 0.001
No	187 (67.8)	576 (66.1)	115 (71.0)	111 (61.7)	895 (68.3)	317 (79.4)	
Yes	89 (32.2)	296 (33.9)	47 (29.0)	69 (38.3)	416 (31.7)	82 (20.6)	
Alcohol consumption, n (%)							0.008
Never drinkers	181 (65.6)	563 (64.6)	120 (74.1)	119 (66.1)	880 (67.1)	297 (74.4)	
Former drinkers	75 (27.2)	237 (27.2)	37 (22.8)	44 (24.4)	307 (23.4)	76 (19.0)	
Current drinkers	20 (7.2)	71 (8.1)	5 (3.1)	17 (9.4)	124 (9.5)	26 (6.5)	
Depressive symptoms, n (%)							< 0.001
No	260 (94.2)	773 (88.6)	135 (83.3)	139 (77.2)	686 (52.3)	160 (40.1)	
Yes	8 (2.9)	66 (7.6)	17 (10.5)	33 (18.3)	565 (43.1)	208 (52.1)	
BMI, n (%)							< 0.001
Underweight	0 (0.0)	0 (0.0)	0 (0.0)	29 (16.1)	182 (13.9)	62 (15.5)	
Normal	143 (51.8)	491 (56.3)	105 (64.8)	104 (57.8)	704 (53.7)	223 (55.9)	
Overweight	99 (35.9)	285 (32.7)	39 (24.1)	31 (17.2)	308 (23.5)	80 (20.1)	
Obesity	34 (12.3)	96 (11.0)	18 (11.1)	16 (8.9)	117 (8.9)	34 (8.5)	
Number of comorbidities, n (%)							< 0.001
0	93 (33.7)	256 (29.4)	43 (26.5)	75 (41.7)	325 (24.8)	75 (18.8)	
1	102 (37.0)	265 (30.4)	65 (40.1)	52 (28.9)	367 (28.0)	135 (33.8)	
≥ 2	81 (29.3)	351 (40.3)	54 (33.3)	53 (29.4)	619 (47.2)	189 (47.4)	
Falls^*^, n (%)							0.031
No	245 (88.8)	718 (82.3)	132 (81.5)	154 (85.6)	1067 (81.4)	317 (79.4)	
Yes	31 (11.2)	154 (17.7)	30 (18.5)	26 (14.4)	244 (18.6)	82 (20.6)	
Fall-induced injuries^*^, n (%)							0.053
0	259 (93.8)	810 (92.9)	146 (90.1)	169 (93.9)	1193 (91.0)	357 (89.5)	
1	15 (5.4)	55 (6.3)	10 (6.2)	7 (3.9)	86 (6.6)	30 (7.5)	
≥ 2	2 (0.7)	7 (0.8)	6 (3.7)	4 (2.2)	32 (2.4)	12 (3.0)	

*: Data from wave 2013

Table 2 presents the results of the longitudinal GEE model for the association between cognitive status, frailty, and CF, and risk of falls and fall-induced injuries. In Model 1, the risk of falls was higher in the SCD, MCI, RCF, and PRCF groups than in the Healthy group. In Model 2, after adjusting for age and sex, the PRCF (OR = 1.883, 95% CI: 1.529–2.377) and RCF (OR = 1.624, 95% CI: 1.294–1.901) groups were associated with a significantly increased fall risk compared to the Healthy group, while the SCD, MCI, and Frailty groups were not. After further adjustments for all covariates in Model 3, the participants in the PRCF group (OR = 1.442, 95% CI: 1.179–1.922) were at a higher risk of falls than those in the RCF group (OR = 1.302, 95% CI: 1.053–1.593), with no significant effect on fall risk observed in the SCD, MCI, or Frailty groups. The results for fall-induced injuries were similar, except for the RCF group (OR = 1.268, 95% CI: 0.880–1.826) in Model 3. Additional details are provided in Supplementary Tables S1 and S2. The overall interaction between time and group was not statistically significant (Supplementary Table S3). The interaction analyses indicated no multiplicative (OR *=* 0.952, 95% CI: 0.618–1.468) or additive interaction effects of cognitive impairment or frailty on fall events (RERI=-0.043, 95% CI: [-0.495, 0.409]; AP=-0.035, 95% CI: [-0.400, 0.329]; S *=* 0.840, 95% CI: [0.172–4.095]).

**Table 2:** Associations between cognitive status, frailty, and CF, and risk of falls and fall-induced injuries

Group	Model 1		Model 2		Model 3	
	OR (95% CI)	*P*-value	OR (95% CI)	*P*-value	OR (95% CI)	*P*-value
Falls						
Healthy	Ref.		Ref.		Ref.	
SCD	**1.227 (1.000–1.505)**	**0.039**	1.223 (0.999–1.498)	0.057	1.161 (0.967–1.471)	0.202
MCI	**1.436 (1.069–1.929)**	**0.019**	1.322 (0.985–1.775)	0.082	1.235 (0.975–1.807)	0.226
Frailty	1.249 (0.953–1.639)	0.222	1.185 (0.907–1.548)	0.257	1.104 (0.818–1.434)	0.528
RCF	**1.659 (1.367–2.013)**	**< 0.001**	**1.624 (1.294–1.901)**	**< 0.001**	**1.302 (1.053–1.593)**	**0.022**
PRCF	**2.201 (1.769–2.740)**	**< 0.001**	**1.883 (1.529–2.377)**	**< 0.001**	**1.442 (1.179–1.922)**	**0.008**
Fall-induced injuries						
Healthy	Ref.		Ref.		Ref.	
SCD	1.050 (0.752–1.505)	0.773	1.038 (0.999–1.498)	0.825	0.929 (0.671–1.287)	0.659
MCI	**1.604 (1.069–1.929)**	**0.027**	1.487 (0.985–1.775)	0.068	1.346 (0.864–2.098)	0.188
Frailty	1.336 (0.953–1.639)	0.283	1.311 (0.907–1.548)	0.315	1.204 (0.713–2.033)	0.488
RCF	**1.822 (1.367–2.013)**	**0.005**	**1.745 (1.116–2.729)**	**0.015**	1.268 (0.880–1.826)	0.203
PRCF	**2.382 (1.769–2.740)**	**< 0.001**	**2.110 (1.529–2.377)**	**< 0.001**	**1.480 (1.011–2.168)**	**0.044**

Notes: Model 1: no adjustment; Model 2: adjusted for age and sex; Model 3: Model 2 + residential area, educational level, marital status, sleep duration, smoking behavior, alcohol consumption, depressive symptoms, BMI, and number of comorbidities

**Fig. 2:**
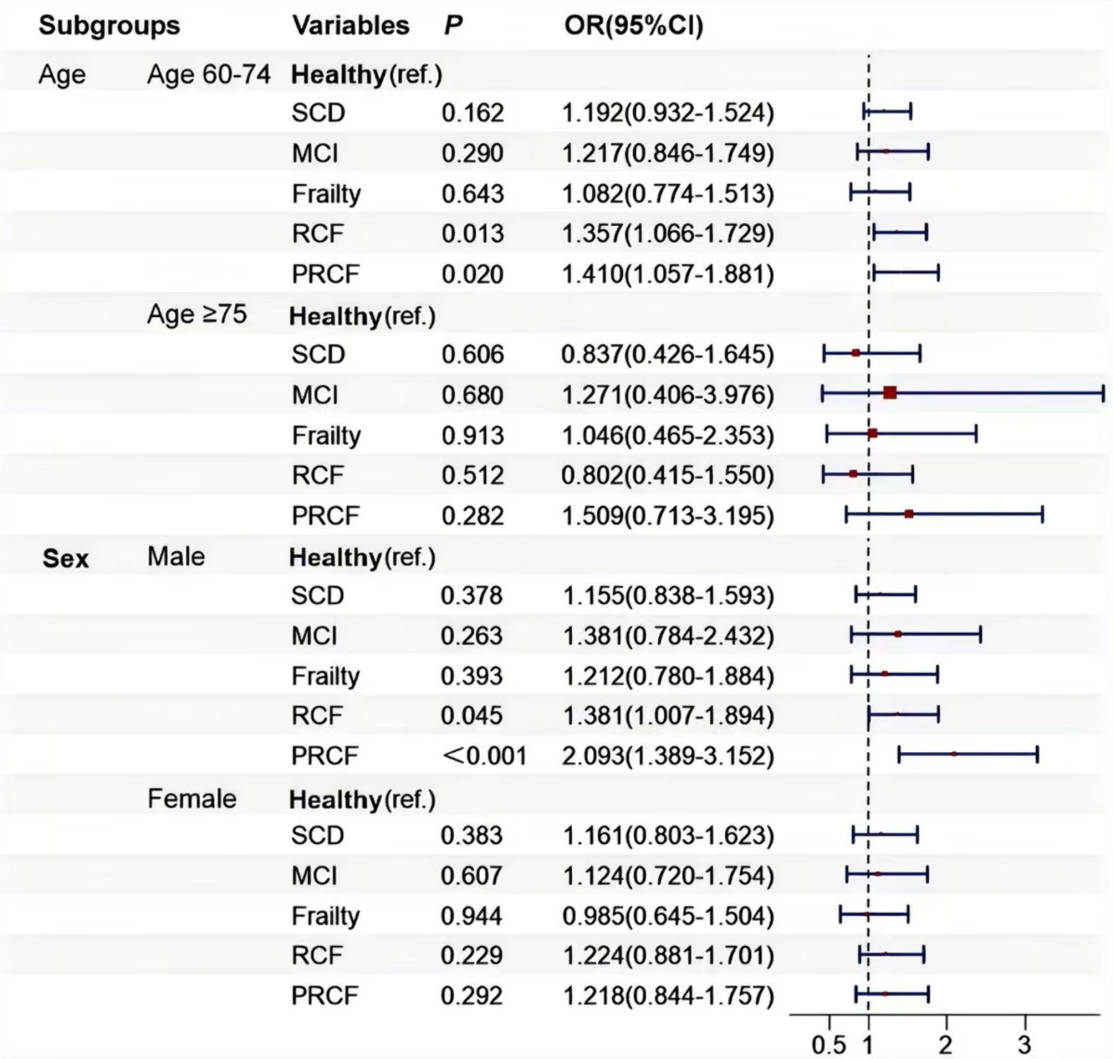
Associations between cognitive status, frailty, CF, and fall risk in different age and sex subgroups. The models were adjusted for age, sex, residential area, educational level, marital status, sleep duration, smoking behavior, alcohol consumption, depression symptoms, BMI, and number of comorbidities

Figure 2 presents the results of the subgroup analysis of the association of cognitive status, frailty, and CF with risk of falls in the different age and sex subgroups based on Model 3. Interactions between age and sex were not statistically significant (*P* = 0.287 and *P* = 0.064, respectively). The results of the sensitivity analysis were consistent with the main findings (Supplementary Tables S4 and S5).

## Discussion

In this longitudinal analysis, we investigated whether RCF and PRCF influence the prevalence of falls in community-dwelling adults aged older than 60 years in China. We found that the risk of falls was greater in RCF and PRCF than in cognitive impairment or frailty alone, with PRCF being associated with a higher risk than RCF. These results indicate that early screening for cognitive impairment and frailty is a necessary strategy to prevent falls in older adults.

Our study differs from existing studies in that we investigated the association between CF and fall occurrence using data from individuals with only cognitive impairment or only frailty as controls in addition to those from a Healthy group. Moreover, we categorized participants with CF into RCF and PRCF, based on the study by Ruan [20]. To assess the effects of different levels of cognitive impairment on falls, we further divided patients with cognitive impairment into SCD and MCI groups. The results showed that CF significantly increased fall risk compared to either cognitive impairment or frailty alone, aligning with findings from previous studies. In one cross-sectional study, Chen et al. reported that CF increased fall risk compared to cognitive impairment or frailty alone [42]. Another study found that participants with CF had a higher risk of falling than healthy individuals, while those with only cognitive impairment and frailty were at a lower risk [19]. In our longitudinal analysis, we similarly found that participants with PRCF had a higher fall risk than participants with RCF, while both groups were associated with an increased fall risk compared with the SCD, MCI, or frailty alone groups. In contrast, Ma et al. reported that the risk of falls was higher in adults with frailty than in those with CF [18]. This discrepancy may be due to differences in the assessment methods for cognitive function, population compositions, and sample sources. Indeed, prior studies have shown that cognitive impairment is often accompanied by neurotransmitter imbalance, brain structural changes (such as atrophy of the hippocampus and prefrontal cortex), and impaired function of the cerebellum and basal ganglia, which affect the processing and transmission of information and, subsequently, gait coordination [43, 44]. Frailty can lead to muscle atrophy and strength decline, decreased bone mineral density, and degradation of neuromuscular function, which directly impacts gait stability. The coexistence of frailty and cognitive impairment may lead to accumulation of the respective pathophysiological changes, thereby further increasing the risk of falls. These findings emphasize the need for early screening for CF to prevent falls in older adults.

We found no multiplicative or additive interaction effects between cognitive impairment and frailty on falls, suggesting that the observed fall risk may simply result from the accumulation of risks associated with cognitive impairment and frailty rather than an interaction between them. The subgroup analysis also revealed no interaction of either age or sex with group, indicating that the effect of CF on falls did not differ across age or sex subgroups. To assess the robustness of our findings, we conducted a sensitivity analysis by combining RCF and PRCF into a single CF category. CF was shown to increase the risk of falls, with no associations found for MCI, SCD, or frailty, aligning with the main findings. Additionally, we analyzed the number of fall-induced injuries, defined as falls serious enough to require medical treatment. Given that most participants did not report any injuries, this variable was exclusively used in the GEE model, and the results aligned with those of our main analyses, reinforcing the reliability of our findings.

This study has some limitations. First, most of the data were self-reported by the participants. Therefore, we cannot exclude recall bias, particularly regarding variables such as fall-induced injuries. To address this concern, we designated two outcome variables and conducted sensitivity analyses using different definitions of CF, which yielded results similar to our main findings, thereby demonstrating robustness and reliability. Second, several factors related to fall risk were missing in CHARLS. Future studies should investigate additional relevant factors, including history of recurrent falls, to provide a more comprehensive understanding of fall risk. Third, the absence of biological sample indicators in the CHARLS dataset may have influenced the definitions of RCF and PRCF. Lastly, given the current unclear definition of SCD, a relatively large percentage of our population fell into the SCD category, which may have led to overestimation or underestimation of our findings. The reported results require further verification in a large population. Overall, we used a method based on HRS to assess cognitive function and defined frailty using the PFP framework, which has been widely recognized in previous studies, ensuring the accuracy of our results. We acknowledge that incorporating biological indicators to refine the definitions of RCF and PRCF would enhance the robustness of the findings.

## Conclusions

This longitudinal study examined the effects of RCF and PRCF on fall risk in community-dwelling adults older than 60 years in China. We found that RCF and PRCF were associated with a higher risk of falls than either cognitive impairment or frailty alone, with the risk being greater in PRCF than in RCF. Therefore, it is necessary to conduct early screening and assessment of cognitive function and frailty to identify individuals at risk for falls in this population.

## Electronic supplementary material

Below is the link to the electronic supplementary material.

Supplementary Material 1

## Data Availability

The data that support the findings of this study are available in the China Health and Retirement Longitudinal Study (CHARLS) repository, http://charls.pku.edu.cn.

## Abbreviations

CF: Cognitive frailty
RCF: Reversible cognitive frailty
PRCF: Potentially reversible cognitive frailty
SCD: Subjective cognitive decline
MCI: Mild cognitive impairment
CHARLS: China health and retirement longitudinal study
PFP: The Fried’s physical frailty phenotype
BMI: Body mass index
CES-D: Center for epidemiological studies depression
HRS: The American health and retirement study
AACD: Aging-associated cognitive decline
SD: Standard deviation
GEE: Generalized estimating equation
OR: Odds ratio
CI: Confidence interval
RERI: Relative excess risk
AP: Attributable proportion
S: Synergy index
