# Supplementary material for: Associations between reversible and potentially reversible cognitive frailty and falls in community-dwelling older adults in China: a longitudinal study
Source: BMC Geriatr. 2025 Apr 5;25:224. doi: 10.1186/s12877-025-05872-2 (PMC11971774; doi:10.1186/s12877-025-05872-2)
Supplement: Supplementary file 1 — Supplementary Material 1 [file 12877_2025_5872_MOESM1_ESM.docx]

**Supplementary materials**

**Table S1** Associations between cognitive status, frailty, and CF, and the risk of falls

| Variables | Model 1 | | Model 2 | | Model 3 | |
| --- | --- | --- | --- | --- | --- | --- |
|  | *OR*(95%*CI*) | *P*-value | *OR*(95%*CI*) | *P*-value | *OR*(95%*CI*) | *P*-value |
| Group |  |  |  |  |  |  |
| Healthy | Ref. |  | Ref. |  | Ref. |  |
| SCD | **1.227(1.000-1.505)** | **0.039** | 1.223(0.999-1.498) | 0.057 | 1.161(0.967-1.471) | 0.202 |
| MCI | **1.436(1.069-1.929)** | **0.019** | 1.322(0.985-1.775) | 0.082 | 1.235(0.975-1.807) | 0.226 |
| Frailty | 1.249(0.953-1.639) | 0.222 | 1.185(0.907-1.548) | 0.257 | 1.104(0.818-1.434) | 0.528 |
| RCF | **1.659(1.367-2.013)** | **<0.001** | **1.624(1.294-1.901)** | **<0.001** | **1.302(1.053-1.593)** | **0.022** |
| PRCF | **2.201(1.769-2.740)** | **<0.001** | **1.883(1.529-2.377)** | **<0.001** | **1.442(1.179-1.922)** | **0.008** |
| Age group |  |  |  |  |  |  |
| 60-75 |  |  | Ref. |  | Ref. |  |
| ≥75 |  |  | 1.115(0.990-1.255) | 0.012 | 1.260(1.047-1.517) | 0.014 |
| Sex |  |  |  |  |  |  |
| Male |  |  | Ref. |  | Ref. |  |
| Female |  |  | 1.576(1.426-1.741) | <0.001 | 1.474(1.292-1.681) | <0.001 |
| Residential area |  |  |  |  |  |  |
| Rural |  |  |  |  | Ref. |  |
| Urban |  |  |  |  | 0.994(0.861-1.149) | 0.467 |
| Educational level |  |  |  |  |  |  |
| Illiteracy |  |  |  |  | Ref. |  |
| Elementary school |  |  |  |  | 1.018(0.900-1.152) | 0.959 |
| Middle school |  |  |  |  | 0.943(0.785-1.133) | 0.487 |
| College or above |  |  |  |  | 0.636(0.377-1.074) | 0.141 |
| Marital status |  |  |  |  |  |  |
| Married |  |  |  |  | Ref. |  |
| Others |  |  |  |  | 1.083(0.956-1.226) | 0.221 |
| Sleep duration |  |  |  |  |  |  |
| <6h |  |  |  |  | Ref. |  |
| 6-9h |  |  |  |  | 0.878(0.757-0.944) | 0.034 |
| ≥9h |  |  |  |  | 0.801(0.637-1.008) | 0.059 |
| Smoking behavior |  |  |  |  |  |  |
| No |  |  |  |  | Ref. |  |
| Yes |  |  |  |  | 1.466(1.300-1.653) | <0.001 |
| Alcohol consumption |  |  |  |  |  |  |
| Never-drinkers |  |  |  |  | Ref. |  |
| Former-drinkers |  |  |  |  | 1.027(0.899-1.172) | 0.889 |
| Current-drinkers |  |  |  |  | 1.144(0.949-1.380) | 0.531 |
| Depressive symptoms |  |  |  |  |  |  |
| No |  |  |  |  | Ref. |  |
| Yes |  |  |  |  | 1.083(0.956-1.226) | 0.221 |
| BMI |  |  |  |  |  |  |
| Underweight |  |  |  |  | Ref. |  |
| Normal |  |  |  |  | 1.212(0.860-1.469) | 0.199 |
| Overweight |  |  |  |  | 1.268(0.923-1.559) | 0.203 |
| Obese |  |  |  |  | 1.320(0.930-1.690) | 0.383 |
| Number of comorbidities |  |  |  |  |  |  |
| 0 |  |  |  |  | Ref. |  |
| 1 |  |  |  |  | 1.107(0.950-1.290) | 0.194 |
| ≥2 |  |  |  |  | 1.343(1.161-1.554) | <0.001 |

**Table S2** Associations between cognitive status, frailty, and CF, and the risk of fall-induced injuries

| Variables | Model 1 | | Model 2 | | Model 3 | |
| --- | --- | --- | --- | --- | --- | --- |
|  | *OR*(95%*CI*) | *P*-value | *OR*(95%*CI*) | *P*-value | *OR*(95%*CI*) | *P*-value |
| Group |  |  |  |  |  |  |
| Healthy | Ref. |  | Ref. |  | Ref. |  |
| SCD | 1.050(0.752-1.505) | 0.773 | 1.038(0.999-1.498) | 0.825 | 0.929(0.671-1.287) | 0.659 |
| MCI | **1.604(1.069-1.929)** | **0.027** | 1.487(0.985-1.775) | 0.068 | 1.346(0.864-2.098) | 0.188 |
| Frailty | 1.336(0.953-1.639) | 0.283 | 1.311(0.907-1.548) | 0.315 | 1.204(0.713-2.033) | 0.488 |
| RCF | **1.822(1.367-2.013)** | **0.005** | **1.745(1.116-2.729)** | **0.015** | 1.268(0.880-1.826) | 0.203 |
| PRCF | **2.382(1.769-2.740)** | **<0.001** | **2.110(1.529-2.377)** | **<0.001** | **1.480(1.011-2.168)** | **0.044** |
| Age group |  |  |  |  |  |  |
| 60-75 |  |  | Ref. |  | Ref. |  |
| ≥75 |  |  | 1.455(1.066-1.987) | 0.015 | 1.457(1.022-2.078) | 0.037 |
| Sex |  |  |  |  |  |  |
| Male |  |  | Ref. |  | Ref. |  |
| Female |  |  | 1.576(1.000-2.079) | 0.050 | 1.230(0.657-2.303) | 0.517 |
| Residential area |  |  |  |  |  |  |
| Rural |  |  |  |  | Ref. |  |
| Urban |  |  |  |  | 0.994(0.861-1.149) | 0.333 |
| Educational level |  |  |  |  |  |  |
| Illiteracy |  |  |  |  | Ref. |  |
| Elementary school |  |  |  |  | 1.018(0.900-1.152) | 0.563 |
| Middle school |  |  |  |  | 0.943(0.785-1.133) | 0.229 |
| College or above |  |  |  |  | 0.636(0.377-1.074) | 0.012 |
| Marital status |  |  |  |  |  |  |
| Married |  |  |  |  | Ref. |  |
| Others |  |  |  |  | 1.083(0.956-1.226) | 0.733 |
| Sleep duration |  |  |  |  |  |  |
| <6h |  |  |  |  | Ref. |  |
| 6-9h |  |  |  |  | 0.845(0.757-0.944) | 0.057 |
| ≥9h |  |  |  |  | 0.769(0.624-0.948) | 0.054 |
| Smoking behavior |  |  |  |  |  |  |
| No |  |  |  |  | Ref. |  |
| Yes |  |  |  |  | 0.995(0.875-1.132) | 0.603 |
| Alcohol consumption |  |  |  |  |  |  |
| Never-drinkers |  |  |  |  | Ref. |  |
| Former-drinkers |  |  |  |  | 0.848(0.899-1.172) | 0.375 |
| Current-drinkers |  |  |  |  | 0.847(0.949-1.380) | 0.531 |
| Depressive symptoms |  |  |  |  |  |  |
| No |  |  |  |  | Ref. |  |
| Yes |  |  |  |  | 1.466(1.300-1.653) | <0.001 |
| BMI |  |  |  |  |  |  |
| Underweight |  |  |  |  | Ref. |  |
| Normal |  |  |  |  | 1.212(1.000-1.469) | 0.511 |
| Overweight |  |  |  |  | 1.268(1.032-1.559) | 0.558 |
| Obese |  |  |  |  | 1.320(1.032-1.690) | 0.946 |
| Number of comorbidities |  |  |  |  |  |  |
| 0 |  |  |  |  | Ref. |  |
| 1 |  |  |  |  | 1.292(1.029-1.622) | 0.028 |
| ≥2 |  |  |  |  | 1.457(1.022-2.078) | <0.001 |

**Table S3** Analysis of the interactions between cognitive status, frailty, and CF, and time

| Variables | Wave | Falls | | Fall-induced injuries | |
| --- | --- | --- | --- | --- | --- |
|  |  | *OR*(95%*CI*) | *P*-value | *OR*(95%*CI*) | *P*-value |
| Healthy*time | Health*wave 2013 | Ref. |  | Ref. |  |
|  | Health*wave 2015 | 1.038(0.657-1.640) | 0.873 | 1.000(0.562-1.779) | 1.000 |
|  | Health*wave 2018 | 1.653(0.993-2.750) | 0.053 | 1.278(0.647-2.524) | 0.480 |
|  | Health*wave 2020 | 1.742(1.065-2.850) | 0.027 | 1.611(0.838-3.099) | 0.153 |
| SCD*time | SCD*wave 2013 | 1.561(1.023-2.383) | 0.039 | 1.227(0.681-2.210) | 0.496 |
|  | SCD*wave 2015 | 1.510(0.988-2.307) | 0.057 | 1.022(0.577-1.812) | 0.940 |
|  | SCD*wave 2018 | 1.587(1.039-2.424) | 0.032 | 1.242(0.705-2.190) | 0.453 |
|  | SCD*wave 2020 | 1.587(1.040-2.422) | 0.032 | 1.038(0.590-1.825) | 0.897 |
| MCI*time | MCI*wave 2013 | 1.506(0.855-2.652) | 0.156 | 2.143(0.981-4.679) | 0.056 |
|  | MCI*wave 2015 | 1.440(0.812-2.552) | 0.212 | 1.401(0.634-3.096) | 0.405 |
|  | MCI*wave 2018 | 2.083(1.214-3.574) | 0.008 | 1.813(0.909-3.617) | 0.091 |
|  | MCI*wave 2020 | 1.643(0.935-2.884) | 0.084 | 1.154(0.564-2.359) | 0.695 |
| Frailty*time | Frailty*wave 2013 | 1.182(0.660-2.118) | 0.574 | 1.948(0.710-5.343) | 0.196 |
|  | Frailty*wave 2015 | 1.360(0.776-2.386) | 0.283 | 1.246(0.527-2.949) | 0.616 |
|  | Frailty*wave 2018 | 2.018(1.183-3.444) | 0.010 | 1.636(0.775-3.452) | 0.196 |
|  | Frailty*wave 2020 | 1.422(0.806-2.508) | 0.224 | 1.013(0.413-2.483) | 0.978 |
| RCF*time | RCF*wave 2013 | 1.383(0.915-2.089) | 0.124 | 1.396(0.799-2.440) | 0.242 |
|  | RCF*wave 2015 | 1.844(1.226-2.775) | 0.003 | 1.446(0.838-2.498) | 0.186 |
|  | RCF*wave 2018 | 1.965(1.306-2.958) | 0.001 | 2.186(0.935-5.114) | 0.071 |
|  | RCF*wave 2020 | 1.836(1.220-2.764) | 0.004 | 1.236(0.710-2.151) | 0.453 |
| PRCF*time | PRCF*wave 2013 | 1.394(0.871-2.229) | 0.166 | 1.558(0.819-2.964) | 0.177 |
|  | PRCF*wave 2015 | 1.923(1.216-3.042) | 0.005 | 1.583(0.864-2.902) | 0.137 |
|  | PRCF*wave 2018 | 2.267(1.438-3.574) | <0.001 | 2.086(1.155-3.766) | 0.015 |
|  | PRCF*wave 2020 | 2.237(1.420-3.525) | 0.001 | 1.985(1.092-3.609) | 0.025 |
| *P*-group |  | 0.073 | | 0.032 | |
| *P*-time |  | <0.001 | | 0.099 | |
| *P*-interaction |  | 0.286 | | 0.750 | |

Notes: Model was adjusted for age, sex, residential area, educational level, marital status, sleep duration, smoking behavior, alcohol consumption, depressive symptoms, BMI, number of comorbidities. *P*-group is the *P*-value for the effect of cognitive frailty on fall. *P*-time is the *P*-value for the effect of cognitive frailty on Fall-induced injuries

**Table S4** Sensitivity analysis of associations between cognitive status, frailty, and CF, and the risk of falls

| Variables | Model 1 | | Model 2 | | Model 3 | |
| --- | --- | --- | --- | --- | --- | --- |
|  | *OR*(95%*CI*) | *P*-value | *OR*(95%*CI*) | *P*-value | *OR*(95%*CI*) | *P*-value |
| Group |  |  |  |  |  |  |
| Healthy | Ref. |  | Ref. |  | Ref. |  |
| SCD | **1.269(1.013-1.589)** | **0.039** | 1.237(0.990-1.546) | 0.062 | 1.158(0.921-1.457) | 0.209 |
| MCI | **1.442(1.040-1.998)** | **0.028** | 1.281(0.924-1.777) | 0.137 | 1.220(0.868-1.716) | 0.252 |
| Frailty | 1.219(0.906-1.638) | 0.190 | 1.192(0.890-1.597) | 0.239 | 1.100(0.810-1.495) | 0.542 |
| CF | **1.806(1.464-2.228)** | **<0.001** | **1.668(1.354-2.056)** | **<0.001** | **1.324(1.059-1.657)** | **0.014** |
| Age group |  |  |  |  |  |  |
| 60-75 |  |  | Ref. |  | Ref. |  |
| ≥75 |  |  | 1.221(1.030-1.447) | 0.022 | 1.250(1.039-1.504) | 0.018 |
| Sex |  |  |  |  |  |  |
| Male |  |  | Ref. |  | Ref. |  |
| Female |  |  | 1.612(1.445-1.798) | <0.001 | 1.488(1.284-1.724) | <0.001 |
| Residential area |  |  |  |  |  |  |
| Rural |  |  |  |  | Ref. |  |
| Urban |  |  |  |  | 0.941(0.799-1.108) | 0.464 |
| Educational level |  |  |  |  |  |  |
| Illiteracy |  |  |  |  | Ref. |  |
| Elementary school |  |  |  |  | 0.986(0.864-1.125) | 0.829 |
| Middle school |  |  |  |  | 0.910(0.748-1.108) | 0.349 |
| College or above |  |  |  |  | 0.602(0.316-1.148) | 0.124 |
| Marital status |  |  |  |  |  |  |
| Married |  |  |  |  | Ref. |  |
| Others |  |  |  |  | 1.091(0.949-1.254) | 0.222 |
| Sleep duration |  |  |  |  |  |  |
| <6h |  |  |  |  | Ref. |  |
| 6-9h |  |  |  |  | 0.877(0.777-0.990) | 0.033 |
| ≥9h |  |  |  |  | 0.804(0.639-1.011) | 0.062 |
| Smoking behavior |  |  |  |  |  |  |
| No |  |  |  |  | Ref. |  |
| Yes |  |  |  |  | 1.018(0.882-1.176) | 0.805 |
| Alcohol consumption |  |  |  |  |  |  |
| Never-drinkers |  |  |  |  | Ref. |  |
| Former-drinkers |  |  |  |  | 0.991(0.855-1.147) | 0.901 |
| Current-drinkers |  |  |  |  | 1.066(0.870-1.305) | 0.539 |
| Depressive symptoms |  |  |  |  |  |  |
| No |  |  |  |  | Ref. |  |
| Yes |  |  |  |  | 1.361(1.190-1.556) | <0.001 |
| BMI |  |  |  |  |  |  |
| Underweight |  |  |  |  | Ref. |  |
| Normal |  |  |  |  | 1.148(0.928-1.420) | 0.203 |
| Overweight |  |  |  |  | 1.155(0.919-1.451) | 0.216 |
| Obese |  |  |  |  | 1.124(0.857-1.474) | 0.400 |
| Number of comorbidities |  |  |  |  |  |  |
| 0 |  |  |  |  | Ref. |  |
| 1 |  |  |  |  | 1.111(0.954-1.295) | 0.176 |
| ≥2 |  |  |  |  | 1.347(1.164-1.558) | <0.001 |

**Table S5** Sensitivity analysis of associations between cognitive status, frailty, and CF, and the risk of fall-induced injuries

| Variables | Model 1 | | Model 2 | | Model 3 | |
| --- | --- | --- | --- | --- | --- | --- |
|  | *OR*(95%*CI*) | *P*-value | *OR*(95%*CI*) | *P*-value | *OR*(95%*CI*) | *P*-value |
| Group |  |  |  |  |  |  |
| Healthy | Ref. |  | Ref. |  | Ref. |  |
| SCD | 1.085(0.771-1.528) | 0.639 | 1.064(0.759-1.491) | 0.720 | 0.923(0.663-1.284) | 0.632 |
| MCI | **1.573(1.045-2.367)** | **0.030** | 1.424(0.938-2.162) | 0.097 | 1.307(0.829-2.062) | 0.249 |
| Frailty | 1.314(0.788-2.192) | 0.295 | 1.274(0.764-2.125) | 0.354 | 1.188(0.701-2.013) | 0.523 |
| CF | **1.942(1.362-2.769)** | **<0.001** | **1.798(1.219-2.653)** | **0.003** | **1.312(0.916-1.879)** | **0.138** |
| Age group |  |  |  |  |  |  |
| 60-75 |  |  | Ref. |  | Ref. |  |
| ≥75 |  |  | 1.379(1.032-1.842) | 0.030 | 1.410(0.980-2.027) | 0.064 |
| Sex |  |  |  |  |  |  |
| Male |  |  | Ref. |  | Ref. |  |
| Female |  |  | 1.499(1.024-2.197) | 0.038 | 1.160(0.564-2.388) | 0.687 |
| Residential area |  |  |  |  |  |  |
| Rural |  |  |  |  | Ref. |  |
| Urban |  |  |  |  | 1.615(0.639-4.081) | 0.311 |
| Educational level |  |  |  |  |  |  |
| Illiteracy |  |  |  |  | Ref. |  |
| Elementary school |  |  |  |  | 1.036(0.850-1.262) | 0.727 |
| Middle school |  |  |  |  | 0.650(0.361-1.171) | 0.152 |
| College or above |  |  |  |  | 0.178(0.049-0.643) | 0.008 |
| Marital status |  |  |  |  |  |  |
| Married |  |  |  |  | Ref. |  |
| Others |  |  |  |  | 0.943(0.744-1.196) | 0.630 |
| Sleep duration |  |  |  |  |  |  |
| <6h |  |  |  |  | Ref. |  |
| 6-9h |  |  |  |  | 0.756(0.564-1.015) | 0.063 |
| ≥9h |  |  |  |  | 0.708(0.492-1.019) | 0.063 |
| Smoking behavior |  |  |  |  |  |  |
| No |  |  |  |  | Ref. |  |
| Yes |  |  |  |  | 0.852(0.516-1.406) | 0.531 |
| Alcohol consumption |  |  |  |  |  |  |
| Never-drinkers |  |  |  |  | Ref. |  |
| Former-drinkers |  |  |  |  | 0.818(0.545-1.228) | 0.332 |
| Current-drinkers |  |  |  |  | 0.827(0.464-1.473) | 0.518 |
| Depressive symptoms |  |  |  |  |  |  |
| No |  |  |  |  | Ref. |  |
| Yes |  |  |  |  | 1.443(1.026-2.030) | 0.035 |
| BMI |  |  |  |  |  |  |
| Underweight |  |  |  |  | Ref. |  |
| Normal |  |  |  |  | 1.152(0.775-1.713) | 0.483 |
| Overweight |  |  |  |  | 0.886(0.636-1.234) | 0.473 |
| Obese |  |  |  |  | 0.964(0.649-1.432) | 0.856 |
| Number of comorbidities |  |  |  |  |  |  |
| 0 |  |  |  |  | Ref. |  |
| 1 |  |  |  |  | 1.296(1.026-1.638) | 0.030 |
| ≥2 |  |  |  |  | 1.682(1.296-2.182) | <0.001 |
